# Aneurismal Varix of the Popliteal Space—Ligature of the Artery and Vein

**Published:** 1848-02

**Authors:** Daniel Brainard

**Affiliations:** Professor of Surgery in Rush Medical College, Surgeon to the Chicago Hospital, &c.


### ARTICLE VI.

*Aneurismal Varix of the popliteal space—ligature of the artery and vein. Hemorrhage, amputation, recovery.* By Daniel Brainard, M. D., Professor of Surgery in Rush Medical College, Surgeon to the Chicago Hospital, &c.

Enoch Kandall, æt. 21 years, had, in the month of June,

1842, a knife thrust into the popliteal space about two inches above the knee, and one inch within the outer ham string. The cut bled profusely at the time, but the hemorrhage was stopped by compresses and a bandage, and the wound adhered by first intention. At the end of about four weeks he began to feel a "fluttering," on placing his hand upon the part, which gradually increased. At the end of six months, he perceived a tumefaction of the part, and at the same time noticed that the veins were enlarged over the whole member.

In August, 1845, he had a slight scratch on the leg, which ulcerated; and this never healing, nearly the whole surface of the leg became ulcerated. He continued to labor until about a year since, when he was compelled to stop work.

January 24, 1848, it presented the following appearances:

There was a tumor filling the popliteal space and rising above its surface, encroaching upon the inner condyle of the femur. It gradually diminished as it extended upward and inwards till it disappeared about the middle of the thigh, and in like manner extended downward till it was lost in the calf of the leg, being circumscribed or having perceptible limits on the outer and inner sides. The color of the skin was natural; it was soft, elastic, fluctuating, gave a strong vibratory sensation to the touch, and pulsated strongly. It could be, in a great measure, removed by pressure, and diminished in size on raising the member or compressing the femoral artery. The veins over the whole limb, both capilaries and trunks, were greatly enlarged, and upon the inside of the leg was a varicose enlargement of the size of half an egg, which in common with the trunks pulsated strongly.

The young man's health was in other respects pretty good. He had been subject to intermittent fever, but had never experienced, so far as could be discovered by questions, any derangement of the functions resulting from the mingling together of the arterial and venous blood. He was subject to hemorrhage, and had, several times during his life, nearly died of epistaxis.

The ligature of the artery was performed in presence of the class at the Medical College, Wednesday, January 26, 1848, as follows: The femoral artery being compressed where it passes over the pubis, and the member raised so as to allow the blood to flow out of it, a tourniquet was applied upon the thigh, about the middle, so as perfectly to interrupt the circulation. An incision was then made vertically over the middle of the popliteal space, about four inches in length. The sac being opened by this, a gush of blood, judged to be about eight ounces, took place, but in a few seconds the flow entirely ceased. Wiping out the sac with a sponge, a state of things very different from that usually described as constituting aneurismal varix, was found; instead of finding the sac coustituted by the enlarged vein, as is sometimes the case, or being situated between the artery and vein, with an opening into each, as has been found in other cases, there was a common cavity into which all the arteries and veins seemed to open, both above, below, and around; the popliteal artery and vein, as they entered from above, being gradually enlarged for some distance, and a similar diminution taking place as they passed from the sac.

The branches of the popliteal artery were also enlarged where they opened into the sac; at least five orifices were seen there sufficiently large to admit a common sized silver catheter a short distance within them.

Owing to this gradual enlargement of the artery, it was necessary to enlarge the wound upward about two inches, when the trunk being found about two or three times its natural size, it was tied with the vein. The wound was then enlarged downward about an inch, and the artery greatly increased in size but with its coats thin like those of a vein, was, with the vein, also secured. The separating and securing these trunks was a thing of great difficulty, and was effected by passing a silver catheter into them, and raising them from their situations as far as could be done. Having thus secured the vessels above and below, the tourniquet was loosened and for about two minutes no hemorrhage took place, but at length the blood returned in a full current and gushed out of the wound. The hemorrhage was arrested by tightning the tourniquet, and it was then determined to dissect up the sac and pass a ligature around it. This was carefully done, and when completed, the tumor formed by the sac, of the size of a hickory nut, was seen at the bottom of the wound. On again loosening the tourniquet it gradually filled with blood, but did not pulsate. Stitches and adhesive straps were then applied to retain the lips of the wound in contact, and a roller applied from the toes to the upper part of the thigh as tightly, as could well be borne, with compresses over the wound. The tourniquet was then placed loosely upon the thigh, to be tightened in case of necessity, and the patient carried to his lodgings, and placed in bed.

He supported the operation with great fortitude, and although it was long, he was not much depressed when it was finished. There was neither pain nor coldness of the member. In the evening gr. x. of Dover's powder was given.

January 27—Morning. Has slept well ; pulse 100 and strong ; no pain ; gave a cathartic of inf. of senna. Evening : Cathartic operated well ; pulse 110 ; ordered v. s. to 18 oz. with gr. x. of pulv. Doveri.

28. Has slept well ; pulse 120 ; some heat of limb with oozing of serum from the wound. Ordered nit. pot. gr. v. with ant. tart. ¼ gr. every two hours, with evaporating lotions to the member. Evening : Gave the Dover's powder.

29. Has rested well ; pulse 120 ; pain and tension in limb ; roller and compresses removed and re-applied ; treatment continued.

30. Has rested well, and has no pain ; pulse 100 ; swelling subsided ; slight discharging of puriform matter from wound. Nitre and tart. ant. discontinued ; corn meal gruel allowed for diet, and sul. mag. ℥i. was administered. Evening: Salts operated well; gave Dover's powder and resumed the aut. tart. and nit. potash.

31.—Morning. Has slept pretty well; the oozing of serum commences to be yellowish from pus; pulse 110.

At three o'clock P. M., five days after the operation, while the patient was lying pefectly quiet in the bed, a profuse hemorrhage took place from the wound and before it could be arrested thirty ounces of blood were lost; by tightning the tourniquet the bleeding was arrested. On arriving I found him much exhausted. Being desirous of ascertaining the source of the hemorrhage, I loosened the tourniquet, and for a short time no bleeding took place, but at the end of about thirty seconds it commenced in a large, uniform stream of dark colored blood, which was not arrested by compressing the femoral artery at the pubis. This at once showed that the source of the hemorrhage was the sac, and that it was venous in its character. It also showed that amputation was the only remedy for the ligature of the branches entering the sac was impossible, and the principal ones were tied both above and below.

This was accordingly done by the circular method about the middle of the thigh. The fortitude of the patient was great, but the vital powers of the system were much depressed and required stimuli which were freely given.

Feb. 1. Has slept well; pulse 130; no pain; allowed gruel and demulcant drinks, an anodyne at evening.

He continued to go on very well until the 11th February, when as he was lying quietly in bed, a considerable hemorrhage occurred from the stump. On arriving, I found that this had been arrested by compressing the femoral artery, and as it did not return on removing the pressure, a tight roller with compresses and evaporating lotions were applied to the stump, and full doses of acet. of lead and opium given internally. The hemorrhage did not return, and at the present time, March 8, the stump is almost cicatrized.

It may be added that in proportion as the circulation in the member diminished in activity, the tissues shrunk away so as to leave the bone too prominent, and in order to prevent the inconveniences of a conical stump, about two inches of it was removed.

On examining the limb it was found that the artery and vein, both above and below, had been perfectly included in the ligature, but in consequence of the great increase of the collateral branches, there was no coagulum in the femoral artery above the ligature. It was greatly enlarged and tortuous. The vein was greatly enlarged and thick, and full of coagulum. The coats of the artery below the aneurismal were thin like those of a vein, and the vein itself was greatly enlarged, forming sacs of the size of an egg; both artery and vein were filled with coagulum. The ligature of the sac had cut through and offered a large opening from which the blood came. There was no coagulum in the sac, the venous circulation having been actively kept up through it. The mouths opening upon its internal surface were found to be veins.

